# Building the future of UK primary care: expanding roles of general practice nurses and patient perspectives

**DOI:** 10.1186/s12875-026-03235-3

**Published:** 2026-03-17

**Authors:** Nagina Khan, Hussain Ismail, Alison Leary, Imelda McDermott, Stephen Peckham

**Affiliations:** 1 Centre for Health Services Studies, School of Social Sciences, University of Kent, Canterbury, Kent UK; 2 NIHR Allied Research Collaborative Kent, Surrey and Sussex (ARC KSS), Canterbury, Kent UK; 3 NIHR INSIGHT Programme, MSc Applied Health and Care Research, Canterbury, Kent UK; 4 Addington Road Surgery, 77 Addington Road, Canterbury, West Wickham UK; 5 London South Bank University (LSBU), WHO Europe, Royal College of Nursing (RCN), London, SE1 0AA UK; 6 Centre for Primary Care and Health Services Research and Centre for Pharmacy Workforce Studies, University of Manchester, Manchester, UK; 7 Centre for Health Services Studies, University of Kent, NIHR Senior Investigator, Co-Director NIHR ARC Kent Surrey and Sussex, Canterbury, Kent UK

**Keywords:** General practice nurses, Primary care, Patient experience, Patient satisfaction, Leadership, Role development, Multidisciplinary teams

## Abstract

**Background:**

General practice nurses’ (GPNs) in UK primary care have undergone significant role expansion over the past decade, encompassing chronic disease management, independent prescribing, preventive care and patient education. Despite this growth, evidence on patient experiences, satisfaction, and organisational factors supporting these roles remains fragmented.

**Aim:**

This scoping review synthesises evidence on the roles and responsibilities of practice nurses in primary care, patients’ perspectives on their care and the organisational and leadership factors that support effective practice within multidisciplinary teams.

**Methods:**

Following the Arksey and O’Malley framework with Levac et al. enhancements and PRISMA-ScR guidelines, we conducted a comprehensive search of major databases and grey literature sources. English-language studies published between 2010 and 2025, including qualitative, quantitative, and mixed-methods designs, were included if they focused on UK general practice nurses and patient experiences. Data were extracted and synthesised thematically to identify patterns related to nursing roles, patient perceptions, and organisational influences.

**Results:**

General practice nurses deliver a wide range of clinical services and are highly valued by patients for their accessibility, approachability, and continuity. Patient satisfaction is highest when nurses demonstrate person-centred communication, clinical competence, professional autonomy, and strong therapeutic alliances. Effective integration within primary care teams is supported by formal leadership, structured professional development, role clarity, and organisational infrastructure. Persistent barriers include variability in employment conditions and the lack of standardised leadership pathways.

**Conclusions:**

General practice nurses are central to delivering high-quality, patient-centred care in the UK. Strengthening formal leadership, enhancing professional development, clarifying roles through supportive policy frameworks and addressing employment variability are essential to maximise their contributions. Investment in these areas will improve patient outcomes, workforce satisfaction and the long-term sustainability of primary care services.

## Introduction

Over the last two decades, the role of nurses in general practice has expanded substantially. Following the introduction of the Quality and Outcomes Framework (QOF) in 2003, general practice nursing (GPNs) shifted from predominantly administrative and supportive tasks to more complex clinical responsibilities [1]. Research on nursing leadership in primary care is limited, unlike in hospital settings, where its impact on outcomes is well established [2–4]. Nurses now undertake diagnostic assessments, chronic disease management, medication management, and, for those with the appropriate qualifications, independent prescribing. Approximately 1 in 5 general practice appointments are now delivered by nurses [5], highlighting their central role in direct patient care. At the same time, the roles of nursing assistants and healthcare assistants (HCAs) have evolved, taking on duties previously performed by GPNs further reflecting the changing skill mix in primary care. For the purposes of this review, ‘general practice nurses’ (GPNs) are defined as registered nurses working within UK primary care or general practice settings. These nurses provide clinical care, chronic disease management, preventive services, patient education, and contribute to multidisciplinary team (MDT) functioning. ‘Primary care’ refers to first-contact, accessible, continuous and comprehensive care delivered in general practice or community-based settings. The review is limited to the UK because nursing roles, training pathways, funding mechanisms and regulatory frameworks are highly context-specific; the structure of the NHS, Primary Care Networks, and workforce policies creates a distinct environment compared with international healthcare systems. Clear definitions of key terms ensure that findings are interpreted appropriately for UK policy and practice while also providing clarity for an international audience [6].

While Primary Care Networks (PCNs) do not pool practice nursing staff, and most nurses remain employed directly by individual General Practitioner (GP) practices, the PCN structure can facilitate collaboration across practices by supporting shared roles such as the Additional Roles Reimbursement Scheme (ARRS)-funded positions and enabling joint initiatives. Introduced in 2019 as part of the UK government’s commitment to improve access to general practice, ARRS allows PCNs to claim reimbursement for the salaries (and some on costs) of 17 additional multidisciplinary team roles, such as clinical pharmacists, social prescribing link workers and physiotherapists. These roles are intended to complement, rather than replace, existing GPNs and are tailored to meet local population needs. By enabling joint initiatives and shared workforce models, PCNs provide an infrastructure that supports collaborative working and service development across practices.

Although GPNs are not yet routinely deployed across multiple practices, the development of PCNs creates emerging opportunities for greater coordination, shared learning, and future models of working that may extend nursing responsibilities in population health and network-level service delivery. However, despite these structural reforms, GPNs in England are generally employed directly by individual practices rather than under national frameworks such as the Agenda for Change (AfC), which is used in acute and community settings. This creates variability in pay, job security, supervision, and career development. Whereas the introduction the AfC in 2004 provided a single pay structure for all NHS staff except doctors, dentists and very senior managers (VSMs). The aim was to harmonise and modernise pay and conditions, terms of employment and HR policies across the NHS. In addition, formal nursing leadership positions within PCNs are scarce and nurses often rely on GPs or practice managers for management and work allocation [7–9]. While registered nurses remain clinically accountable for their own practice, the absence of embedded nurse leadership limits support for professional development, role integration, and career progression [10, 11]. Fragmented employment arrangements, lack of standardised supervision and differences in role definitions across practices contribute to inconsistencies in nurse experiences and patient care quality.

In Scotland, while many GPNs remain employed by individual GP practices, national policy frameworks such as the Transforming Roles agenda and Health and Social Care Partnership (HSCP)/Health Board governance structures embed nursing leadership, define consistent career pathways, and provide standardised education and professional support across the system [12–14]. This allows for more coordinated deployment of nursing staff, structured supervision and clearer career progression pathways [15]. Nurses are better integrated across practices and community services, which supports continuity of care, interprofessional collaboration and workforce sustainability [16]. Nevertheless, challenges remain at the local level: resource allocation, workforce shortages and variable implementation of leadership structures can affect how nurses experience their roles and the consistency of patient care [17–20]. Across the UK, differing organisational and leadership arrangements, including those in England, Scotland, Wales, and Northern Ireland, shape how nurses enact their roles and deliver care. These systemic differences influence workforce experiences, multidisciplinary team integration, and, potentially, patient outcomes, highlighting the importance of policy-aligned leadership models that support nurse capability and role clarity.

Expanding scope of practice and professional identity has also influenced nursing in primary care. GPNs increasingly perform complex clinical responsibilities, once the domain of GPs, such as prescribing, diagnostic tests and chronic disease management, alongside traditional responsibilities like vaccination and health promotion [21]. These changes highlight the need to consider not only formal practice nursing role competencies defined in training programmes which are the generic, foundational knowledge, skills, and professional attributes required for an entry-level or general role but also ‘role-holder competencies,’ skills, knowledge, and capabilities developed through individual experience and local adaptation in real world context [22]. Differences between role competencies (what a role requires) and role-holder competencies (the skills an individual brings) highlight why standardised job titles do not necessarily reflect actual responsibilities or expertise across settings. In this review, the term GPNs is used as the overarching title, but responsibilities vary depending on experience, local practice context, team dynamics, and management structures. Nurses’ professional identity is shaped by these factors, influencing how they enact roles, collaborate with other professionals, and impact patient care. Professional identity is also informed by regulatory and professional frameworks, including the Nursing and Midwifery Council (NMC) and other relevant governing bodies, which establish standards of practice, education and accountability [23, 24].

Leadership gaps in practice nursing remain a significant barrier. In England, formal nursing leadership within general practice remains limited, with most GPNs employed directly by individual GP practices and often working without structured leadership pathways, hierarchy or professional networks. Although nurses can hold senior roles such as the Primary Care Network (PCN) Clinical Director, this remains uncommon in practice, contributing to a sense of professional isolation and limited strategic influence across networks. Management functions, such as work allocation, appraisal and professional development, are typically performed by GPs or practice managers rather than by GPNs with leadership training [21, 25]. The lack of embedded nurse leaders reduces opportunities for mentorship, advocacy and team cohesion, which can negatively affect staff satisfaction, retention, and patient outcomes. In Scotland, embedding nurse leadership within HSCPs and Health Boards provides more consistent support, supervision, and guidance for GPNs, helping to integrate their roles across practices and community services. Nevertheless, implementation varies, and local challenges, such as staffing shortages, resource limitations, and organisational readiness, continue to affect the effectiveness of nursing leadership in general practice settings.

Patient perspectives and satisfaction are crucial to understanding the impact of nursing roles. Evidence suggests high patient satisfaction when care is relational, continuous, and delivered by competent staff, however, trust and negative perceptions of authority can influence engagement, particularly in areas such as chronic disease management and prescribing [26]. GPNs’ contributions to multidisciplinary teams, care coordination, patient education, and structured reviews enhance service delivery and population health outcomes, highlighting the importance of integrating nursing perspectives into workforce planning and policy development [27].

### Focus and rationale for the scoping review

This review focuses on GPNs to examine the evolving scope of practice, organisational integration, and leadership support. It aims to synthesise evidence on:

1. Roles and responsibilities of GPNs in primary care.
2. Patient views, experiences, and satisfaction with care.
3. Contributions of GPNs to multidisciplinary team (MDT) functioning and integrated care.
4. Organisational and leadership factors influencing role enactment, professional development and workforce sustainability.

By consolidating these insights, the review will inform future research, workforce planning, and policy development, particularly around retention, skill utilisation, and role clarity. Understanding leadership structures, supervision and standardised career pathways is critical for optimising the deployment of GPNs, reducing role ambiguity, and enhancing integration within primary care teams. Evidence on workforce challenges, including high turnover, part-time working patterns and variability in training opportunities, will help policymakers and service planners develop strategies that support sustainable, patient-centred care and maximise the contribution of GPNs across the UK.

## Methods

The primary objective was to map and synthesise the available evidence on GPNs’ roles and patient perspectives on their care provided by GPNs in primary care settings.

### Review questions and specific objectives

5. To identify and describe the roles and responsibilities of GPNs in primary care.
6. To explore patients’ views, experiences and levels of satisfaction with care provided by GPNs.
7. To examine how GPNs contribute to multidisciplinary team (MDT) working in primary care.
8. To identify organisational and leadership factors that facilitate and/or hinder the effective integration of GPNs and their impact on patient care.

These objectives guided our literature search, data extraction and thematic synthesis.

### Scoping review framework

In this review we followed the Arksey and O’Malley framework (2005) [28], enhanced by Levac et al. (2012), [29] and reported according to PRISMA-ScR guidelines [30].

### Eligibility criteria

#### Population

GPNs in primary care and patients receiving their care. While the search strategy captured studies including other primary healthcare nurses, this review focuses specifically on GPNs in UK general practice to reflect the primary care context and maintain relevance to UK workforce policies and patient care delivery.

#### Concept

Roles, responsibilities, scope of practice, patient perspectives, satisfaction, and experiences.

#### Context

General practice/primary care settings in the UK. The review was limited to the UK because nursing roles, training pathways, funding mechanisms and regulatory frameworks are highly context specific. The NHS, Primary Care Networks and workforce policies create a unique environment distinct from international systems. Focusing on UK studies ensures relevance to policy and practice. Included studies reflected both urban and rural practices, and encompassed in-person, remote, and telephone-based care, capturing the diversity of primary care delivery models.

#### Sources

Peer-reviewed qualitative, quantitative and mixed-methods studies; relevant grey literature.

#### Timeframe

2010–2025; English language. Studies from 2010 onward capture when practice nursing roles became more established after the 2004 GMS contract and Quality and Outcomes Framework reforms. By 2010, chronic disease management, structured care pathways, and performance-driven primary care were embedded, supporting clearer role delineation and expanded responsibilities. This period also covers later reforms, including Primary Care Networks (2019) in England and Health and Social Care Partnerships in Scotland, allowing an assessment of contemporary role development.

### Search strategy

Electronic databases searched included MEDLINE (via PubMed), CINAHL, Embase, PsycINFO, Scopus and Web of Science. Grey literature sources included NHS England, the Royal College of Nursing, the Nuffield Trust, The King’s Fund and Department of Health reports.

A structured search strategy was developed using Medical Subject Headings (MeSH) and free-text terms. For example, the MEDLINE (PubMed) search combined terms relating to population, setting and outcomes as follows:

*(“Practice Nurse*” OR “General Practice Nurse*” OR “Primary Care Nurse*” OR “Family Practice Nurse*”).

AND (“role*” OR “scope of practice” OR “expanded role” OR “advanced practice”).

AND (“patient experience” OR “patient satisfaction” OR “patient perspective*”) *

Search terms were adapted as appropriate for each database and reference lists of included studies were searched to identify additional relevant literature.

### Selection of sources and PRISMA flow

**Fig. 1:**
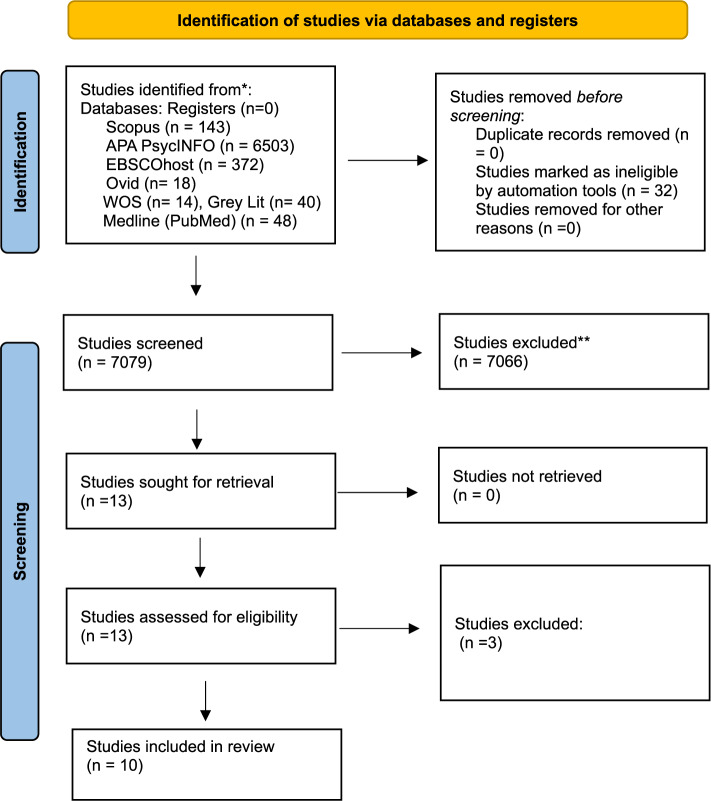
PRISMA 2020 flow diagram

Two reviewers independently screened all titles and abstracts against the inclusion criteria, followed by full text review of potentially relevant studies. Discrepancies at either stage were resolved through discussion and if consensus could not be reached, a third reviewer was consulted to make the final decision. This process ensured a transparent and reproducible selection of studies for inclusion in the review. Please see, Fig. 1. PRISMA 2020 flow diagram for identification of studies [31].

The search identified 7,079 records from electronic databases and grey literature sources. Prior to screening, 32 records were removed after being marked as ineligible by automation tools, with no duplicate records identified. Title and abstract screening was conducted on 7,079 records, of which 7,066 were excluded for not meeting the inclusion criteria. The most common reasons for exclusion were studies conducted outside the UK, focus on non-general practice nursing populations, hospital or community-based settings, absence of patient perspectives, or lack of relevance to nursing roles or scope of practice.

Thirteen reports were sought for retrieval, and all were successfully obtained. Full-text eligibility assessment was conducted for 13 reports, of which two were excluded because they did not report empirical findings relevant to patient perspectives on general practice nursing roles. Eleven studies met the inclusion criteria and were included in the review.

### Data extraction and synthesis

Data were extracted from included studies using a structured template capturing study design, country, population including sample size, GPNs roles, patient perspectives, and key outcomes, directly addressing the review questions on (1) roles and responsibilities of practice nurses, (2) patient experiences and satisfaction, (3) contributions to multidisciplinary team working, and (4) organisational and leadership factors influencing practice. A thematic synthesis was undertaken to identify recurring patterns, relationships, and overarching themes across the included studies. An initial coding framework was developed based on the review questions (roles and responsibilities, patient perspectives, and organisational influences). Data extracted from each study were systematically coded in Microsoft Word using an inductive-deductive approach: pre-defined categories guided analysis, while new codes were generated where findings extended beyond the initial framework.

Codes were compared across studies to identify areas of convergence, divergence, and contextual variation. Related codes were then grouped into higher-order themes through an iterative process of review and refinement. Summary tables were developed to facilitate comparison across study design, setting and key findings. Themes were discussed and refined to ensure they accurately reflected the breadth of evidence and were clearly aligned with the review aims. This approach enabled transparent mapping of the evidence base and identification of patterns and gaps in the literature, consistent with PRISMA-ScR guidance for scoping reviews [31].

## Results

A total of ten studies, published between 2010 and 2025, were included, comprising qualitative, quantitative, mixed-methods, and review designs (see Table 1). Only studies conducted in the UK were included to provide a context-specific understanding of primary care nursing roles and workforce issues. The studies involved patients with chronic diseases, mental health needs, and parents of young children. GPNs roles examined included chronic disease management, independent prescribing, preventive care, triage, and contributions to multidisciplinary teams (MDTs) (see Table 2). Patient perspectives and organisational factors were consistently reported. The findings are presented below, aligned with the review questions.

**Table 1:** Summary of Included Studies

Study	Country	Study Design	Population	GPN Roles/Responsibilities	Patient Perspectives	Key Themes/Findings
Bennett et al., 2013 [32]	UK	Qualitative	Patients with chronic depression; GPNs	Structured chronic disease reviews, care coordination	High satisfaction with continuity; valued relational care	Chronic disease management; relational care; MDT contribution
Hill & Cox, 2013 [33]	UK	Qualitative	Parents of young children	Immunisation, child health checks	Trusted advice from nurses; felt supported	Preventive care; accessibility
Wilson et al., 2012 [34]	UK	Mixed methods	Patients with chronic disease; GPNs	Chronic disease management, health monitoring	Appreciated follow-up and advice; continuity important	GPN expertise, patient trust, continuity
Procter et al., 2013 [34]	UK	Qualitative	Adult patients; GPNs	Chronic disease management, MDT coordination	Positive experience with accessibility and communication	MDT integration; patient-centred care; workload redistribution
Tinelli et al., 2015 [35]	UK	Survey	Patients receiving GPN or pharmacist prescribing	Independent prescribing, patient education	Trusted nurses for prescriptions; valued explanations	Nurse prescribing; patient trust; role recognition
Gerard et al., 2015 [36]	UK	Mixed methods	Patients with long-term conditions	Prescribing, chronic disease management	Satisfaction high; some preference for GP input for complex cases	Nurse competence; patient confidence; role clarity
Calitri et al., 2015 [37]	UK	Quantitative	Adult patients receiving nurse telephone triage	Remote triage, advice	Satisfaction varied; lower for brief, impersonal calls	Access; patient perceptions of communication; remote care challenges
Stenner et al., 2011 [38]	UK	Qualitative	Patients with diabetes	Consultations, prescribing, education	High satisfaction; valued listening and personalised care	Patient-centred care; continuity; trust
Hindi et al., 2019 [39]	UK	Review	Primary care GPNs	Prescribing, chronic disease management, triage	Patients perceived improved access and care quality	Role expansion; workforce efficiency; training needs
Bosley et al., 2021 [40]	UK	Qualitative	Mothers with young children	Child health advice, immunisations	Felt supported; valued approachable care	Relational care; continuity; accessibility

**Table 2:** Synthesis Findings

Theme	Sub-theme	Key Findings
1. Roles and Responsibilities of General Practice Nurses (GPNs)	Chronic Disease Management	GPNs act as case managers in diabetes [38], chronic depression [41], and multiple long-term conditions [34]. Contributions include prescribing, regular monitoring, medication reviews, and structured proactive care
	Prescribing and Autonomy	Independent prescribing improves access, continuity, and skill utilisation [35, 36, 39]. Patients value nurses as ‘expert clinicians’ when they demonstrate prescribing authority and diagnostic expertise [34].
	Preventive and Public Health Roles	GPNs are key in immunisation decision-making [33] and child health support [40]. They provide reassurance, education, and practical support, often acting as the most accessible professional within the GP team
	Triage and Remote Consulting	Nurse-led telephone triage shows mixed patient satisfaction: generally lower than GP contact, but highly valued by patients living further from practices [37].
2. Patient Views, Experiences, and Satisfaction	High Satisfaction and Trust	Consistently high patient satisfaction (> 90%) in prescribing and consultation studies [36, 38, 39]. Trust is enhanced when nurses demonstrate expertise, continuity, and approachability
	Therapeutic Alliance Matters	Strong nurse, patient relationships are central to satisfaction in chronic depression [32] and diabetes management. [38] Dissatisfaction occurs when reviews are procedural, or nurses appear less confident
	Contextual Nuances	Mothers trust GPNs for empathy and reassurance in child health [40]. Parents value GPNs as credible advisers for immunisations [33]. Some patients still prefer GP care when nurse roles seem subordinate [42].
3. Contribution to Multidisciplinary Team (MDT) Working	MDT Roles	GPNs bridge gaps in access and continuity between patients and GPs [33, 40]. They play a central role in multidisciplinary teams (MDTs), facilitating communication, care coordination, and more effective use of staff skills. Evidence from integrated care models indicates that structural and systemic factors, including funding, data systems, and commissioning arrangements, can constrain the full impact of GPNs [42, 43]. While responsibilities such as prescribing and triage help redistribute GP workload, their effective implementation depends on adequate organisational support, targeted training, and clear integration within MDTs [37, 39].
4. Organisational and Leadership Factors		Role enactment was shaped by organisational structures, leadership, and supervision. In England, GPNs are employed by individual practices, with PCNs offering funding (e.g., ARRS) but limited professional oversight. In Scotland, GPNs were also practice-employed but supported by HSCP/Health Board structures that provide clearer leadership and training. Centralised leadership, mentoring and standardised competencies enhance role integration, whereas fragmented management and variable resources limit effectiveness [25, 42, 44].
Barriers and Facilitators to Effective Nursing Roles	Barriers	Lack of competence/confidence in prescribing [39], training gaps, organisational limitations, service cuts [40], stigma in mental-health interventions, fragmented/protocol-driven care [34, 42].
	Facilitators	Organisational support and mentoring, person-centred approaches, continuity of care [38], strong MDT collaboration and visible nurse leadership [32, 34], patient-centred consultation attributes [36].
Synthesis of Key Facilitators and Barriers	Expanded Scope	GPNs increasingly responsible for prescribing, chronic disease management, preventive services, and triage [34–36, 39].
	Patient-Centred Care	Trust and satisfaction highest when GPNs provide holistic, empathetic, and unhurried care [32, 34, 38, 40].
	Conditional Acceptance	Patients are more likely to accept GPN-led care when they perceive nurses as knowledgeable, skilled and confident in their practice, demonstrate continuity, and communicate effectively; otherwise, they may prefer GP-led care [34, 36, 38].
	Systemic Constraints	Structural barriers (training, funding, service cuts) limit GPN impact despite strong patient trust and satisfaction [37, 39].
	Central to MDTs	GPNs act as accessible, trusted professionals who support continuity, bridge gaps between patients and multiple MDT members (GPs, pharmacists, allied health professionals, mental health staff), and relieve workload. Full contribution depends on organisational design, role clarity and integration within the team [32, 34].

Note: In this table, ‘expanded scope’ refers to formally broadened professional responsibilities, ‘expanded role’ refers to tasks beyond traditional duties requiring new skills, and ‘enhanced role’ refers to additional responsibilities within the existing scope of practice

### Review Question 1: Roles and Responsibilities of GPNs

GPNs are increasingly responsible for a wide range of activities, including chronic disease management, independent prescribing, preventive care, triage, and patient education [32, 34, 42]. They often act as case managers for patients with long-term conditions and coordinate care across multidisciplinary teams [34]. Nurse-led interventions redistribute workload from GPs, improve access and support continuity of care. Autonomy and prescribing authority enhance role effectiveness and patient trust.

### Review Question 2: Patient Views, Experiences, and Satisfaction

Patients consistently report high satisfaction with GPN-led care, particularly when consultations are accessible, unhurried and patient-centred [34, 38, 40]. Trust and confidence in nurses increase when they demonstrate expertise, maintain continuity, and engage in strong therapeutic alliances. Some patients still prefer GP-led care for complex issues, but satisfaction with practice nurse led services is higher when nurses provide competence, clarity and tangible support.

### Review Question 3: Contribution to multidisciplinary team (MDT) working

GPNs played a central role in MDT functioning, bridging gaps in access and continuity, and enabling more effective utilisation of staff skills [34]. Evidence suggests that teams with well-integrated nurse-led interventions report higher patient satisfaction and better workflow efficiency. Clear role definitions, professional autonomy and collaborative practice are key enablers.

### Review Question 4: Organisational and leadership factors

#### Employment and oversight structures

- In England, GPNs were primarily employed by individual GP practices, with PCNs providing funding via ARRS but limited direct oversight [32, 34].
- In Scotland, GPNs were also largely practice employed; HSCPs and Health Boards provide professional guidance, training and coordination.

#### Leadership and mentorship

Centralised leadership structures, structured supervision and formal mentoring facilitate role integration and enable nurses to expand responsibilities effectively [34, 36].

#### Organisational support and resource availability

- Adequate organisational support, including protected time, training opportunities, and clear role delineation, enhances role enactment and patient care [34].
- Conversely, fragmented leadership, resource limitations, and unclear reporting lines constrain effectiveness, reduce confidence in autonomy, and limit the ability to participate in MDTs [33].

#### Standardised competencies

Standardised competencies and practice frameworks support consistent care delivery and professional identity development across diverse practice settings [36, 39].

### Synthesis of key facilitators and barriers

Key facilitators include formal leadership pathways, mentoring, professional development, standardised role definitions and supportive policy frameworks. Barriers include variability in employment conditions, inconsistent role clarity and limited local leadership capacity. Addressing these factors is essential to optimise patient outcomes, enhance workforce satisfaction and sustain primary care services.

GPNs in UK primary care deliver expanded clinical services, are highly valued by patients, and play a central role within multidisciplinary [32, 34, 42]. Their effectiveness is influenced by organisational structures, leadership, and opportunities for professional development [33, 38, 40]. Patients report high trust and satisfaction, although acceptance of expanded roles depends on perceived competence, continuity, and quality of interpersonal care [35, 36]. Systemic and organisational constraints such as funding, training, and service design, continue to limit the full realisation of general GPNs contributions [37, 39].

## Discussion

The role of GPNs in primary care has evolved significantly over the past decade, reflecting policy changes, workforce demands and the expansion of clinical responsibilities. The evidence from the scoping review highlights that GPNs now perform a broad spectrum of functions, ranging from chronic disease management and independent prescribing to mental health support, triage, preventative care and patient education [32, 35, 42]. This expansion of scope highlights their pivotal position within primary care, particularly in integrating care within multidisciplinary teams supporting multidisciplinary team (MDT) functioning and maintaining patient-centred services. While the review captures the functional roles of GPNs, there is limited evidence addressing their professional identity, and perceptions of role integration within an expanding multidisciplinary team in primary care. This gap highlights the need for further research exploring how nurses understand and enact their roles in the context of changing organisational structures.

### Expanded clinical roles and patient perspectives

Studies consistently indicate that patients value GPN-led care, especially when it is accessible, continuous and delivered with a patient-centred approach. Patients report high satisfaction with nurse-led prescribing and chronic-disease management, perceiving nurses as approachable, competent and trustworthy [35, 36, 39] This competence is a key contributor to patient safety. Structured interventions, such as proactive care for chronic depression, demonstrate that regular follow-ups and strong therapeutic alliances significantly improve patient engagement and perceived quality of care and safety [32]. Similarly, patients appreciate the clarity and time afforded in nurse consultations, which often contrasts with perceived rushed GP appointments [35].

However, evidence suggests that patient preferences remain nuanced. Some express a baseline preference for GP care, particularly for complex or acute issues, though this can be offset when nurses demonstrate competence, offer patient-centred attention and provide tangible support prescribing [35, 36, 39] Evidence also suggests that accessibility and contextual factors, such as distance from the practice, can influence satisfaction with nurse-led services [37]. Collectively, these findings emphasize the need to embed GPNs within care models that support continuity, accessibility and holistic engagement.

### Leadership and organisational influences

The effective integration of GPNs’ roles is strongly contingent on formal leadership structures [39]. While hospital-based nursing leadership is well-documented as promoting staff satisfaction, retention, and high-quality care [32], primary care settings frequently lack equivalent structures. In our review, several studies reported that GPNs were often line-managed by GPs or practice managers whose focus was primarily clinical and operational, rather than on professional development [33, 34]. These limited opportunities for nurses to develop extended or complex roles and contributed to role ambiguity, workload pressures, and inconsistent support for role expansion.

Our findings also showed that practices with stronger leadership support, for example, clear supervision, structured mentoring, and shared decision-making, enabled nurses to undertake higher-level clinical activities such as prescribing, chronic disease management and triage [35, 36]. These settings also reported better continuity of care and enhanced patient satisfaction [38, 40]. Structured leadership pathways, formal mentoring and targeted training in clinical, operational and policy domains emerged as key enablers, facilitating nurse autonomy, effective MDT collaboration and improved organisational capacity. This evidence highlights the need for investment in leadership development within primary care to fully realise the potential of GPNs and optimise patient outcomes [32–36, 38, 39].

### Standardisation and role clarity

Another critical enabler identified in our review is the need for standardised role definitions. Variation in responsibilities, pay and employment conditions across practices creates inconsistencies in how GPNs operate and are perceived by both colleagues and patients [32, 34, 36]. Clear articulation of competencies, scope of practice and accountability enhances collaboration within multidisciplinary teams (MDTs), reduces role conflict, and supports more consistent delivery of patient-centred care [34, 35, 38]. Standardised frameworks and role clarity also increase patient trust and confidence, as nurses are recognised for their expertise and responsibilities are well understood across the practice and team [33, 40].

### Integration within multidisciplinary teams

GPNs contribute substantially to multidisciplinary team (MDT) functioning by coordinating care, extending access, and bridging gaps between patients and different professional roles, including GPs, pharmacists, health care assistants, and allied health professionals. Studies indicate that teams with integrated nurse-led interventions achieve improved patient satisfaction and more effective utilisation of staff skills [34, 35, 39]. Collaborative practice is essential: nurses with autonomy and prescribing authority are more effective, as patients trust and respond to their expertise when roles are clearly defined, responsibilities are well understood across the team, and organisational support is in place.61 Integration is facilitated by clear communication, standardised competencies, structured supervision, and supportive leadership, ensuring that GPNs can contribute fully to patient-centred care and team efficiency [32].

### Policy and system-level considerations

Differences in organisational structures, such as England’s Primary Care Networks (PCNs) and Scotland’s Health and Social Care Partnerships (HSCPs), shape how nursing roles are supported and coordinated within primary care. While PCNs provide funding streams such as via the Additional Roles Reimbursement Scheme), oversight, and a framework for collaborative working, the employment of GPNs largely remains with individual GP practices. Similarly, in Scotland, most PNs are employed by GP practices, although HSCPs and Health Boards provide leadership, professional guidance, and coordination across practices. Centralised oversight structures can facilitate nursing leadership, access to training, and peer-support networks, helping nurses integrate across practices and community services [25, 44]. Conversely, practices with limited resources or weak local leadership may struggle to support expanded nursing roles, potentially affecting the quality, consistency and reach of nurse-led care.

### Synthesis of evidence

Overall, the scoping review indicates that GPNs deliver high-quality, patient-centred care and are central to multidisciplinary teams (MDTs) in primary care. Key facilitators of effective practice identified in this review include:

- Formal leadership development and mentoring essential for role integration, confidence, and navigating MDT dynamics [32, 33, 36].
- Standardised role definitions and competencies reduce role ambiguity, enhance collaboration, and improve patient trust [34, 35, 38].
- Targeted professional development in clinical, operational, and leadership domains. Supporting extended roles, prescribing, chronic disease management, and preventive care [34, 40].
- Policy frameworks supporting visibility, employment parity, and integration of nursing roles. Ensuring consistent recognition across practices and promote equitable workforce development [37].

Addressing these factors is essential to maximise GPNs’ contribution, improve patient outcomes, and ensure sustainable workforce development in UK primary car.

### Limitations

Only English-language studies, primarily from the UK, were included. While this focus was intentional to capture the unique structural, regulatory, and funding context of UK primary care, including NHS policies, Primary Care Networks (PCNs), and workforce frameworks, it limits the generalisability of findings to other healthcare systems. Most studies focus on general practice, which reflects the main employment setting of GPNs, but this leaves limited insight into wider primary care settings, rural areas, or less-resourced contexts, representing a gap in the evidence. The evidence base is heterogeneous, encompassing qualitative, quantitative, and mixed-methods studies that vary in quality, sample size, and outcome measures, which may affect the consistency and comparability of reported themes, particularly regarding patient satisfaction [35, 42]. Several studies, such as Calitri et al. and Bosley et al., relied on self-reported or retrospective data, introducing potential bias. [37, 40] Despite systematic searches and inclusion of grey literature, relevant unpublished reports may have been missed. These limitations highlight the need for high-quality, longitudinal research to evaluate the impact of expanded nursing roles, leadership development and multidisciplinary team integration on patient outcomes and workforce sustainability in UK primary care.

## Conclusion

As the roles of GPNs continue to evolve in primary care, addressing formal leadership, structured training and role clarity is essential to support their expanding scope. GPNs now deliver complex clinical care, contribute to multidisciplinary teams (MDTs) and provide patient-centred services central to primary care effectiveness.

### Evidence from the literature highlights several key priorities

Formal Leadership Development: Structured leadership pathways, mentoring and defined career progression empower GPNs to influence service delivery, advocate for patients and strengthen team cohesion [45]. Evidence indicates that leadership development improves job satisfaction, retention, decision-making confidence and consistency of care across practices and networks. In its absence, nurses often work in isolation, limiting professional growth and system-level impact [45].

Role Standardisation and Negotiation: Clear definitions of responsibilities, competencies, and scope of practice reduce ambiguity, facilitate MDT integration, and optimise utilisation of nursing skills. Nursing roles are often negotiated between the practice and individual nurses, with tasks shaped by context, experience, and personal capabilities [46, 47]. Recognising both formal competencies and individual skills supports collaboration, patient safety, and continuity of care. Transparent communication within teams enables flexible deployment of nursing expertise across practices and networks.

Targeted Training: Continuous professional development in clinical, operational, and leadership domains equips GPNs to deliver safe, high-quality, patient-centred care, tailored to expanded roles and multi-practice working [45].

Policy Support: National and regional frameworks are needed to address employment disparities, promote professional recognition and strengthen the visibility and authority of GPNs. Variability in pay, terms and professional development opportunities persists, affecting recruitment and retention. Policy interventions supporting fair remuneration, structured career pathways, and professional development enhance team functioning, workforce sustainability, and patient outcomes [48, 49].

Investing in leadership through formal structures that provide opportunities for GPNs to develop, influence decision-making, and participate in governance, along with role clarity, targeted training and aligned policies, will optimise GPNs’ contributions, enhance patient outcomes, support staff satisfaction and retention and foster a sustainable, effective primary care workforce.

## Data Availability

The data supporting the findings of this mixed methods review are derived from publicly available published studies. Extracted data and coding frameworks generated during the review are available from the corresponding author on reasonable request.
